# MicroRNA: Biogenesis, Function and Role in Cancer

**DOI:** 10.2174/138920210793175895

**Published:** 2010-11

**Authors:** Leigh-Ann MacFarlane, Paul R. Murphy

**Affiliations:** Department of Physiology & Biophysics, Faculty of Medicine, Dalhousie University, 5850 College Street, Sir Charles Tupper Medical Building, Halifax, Nova Scotia, B3H 1X5, Canada

**Keywords:** MicroRNA, RNA interference (RNAi), Post-transcriptional gene regulation, Cancer.

## Abstract

MicroRNAs are small, highly conserved non-coding RNA molecules involved in the regulation of gene expression. MicroRNAs are transcribed by RNA polymerases II and III, generating precursors that undergo a series of cleavage events to form mature microRNA. The conventional biogenesis pathway consists of two cleavage events, one nuclear and one cytoplasmic. However, alternative biogenesis pathways exist that differ in the number of cleavage events and enzymes responsible. How microRNA precursors are sorted to the different pathways is unclear but appears to be determined by the site of origin of the microRNA, its sequence and thermodynamic stability. The regulatory functions of microRNAs are accomplished through the RNA-induced silencing complex (RISC). MicroRNA assembles into RISC, activating the complex to target messenger RNA (mRNA) specified by the microRNA. Various RISC assembly models have been proposed and research continues to explore the mechanism(s) of RISC loading and activation. The degree and nature of the complementarity between the microRNA and target determine the gene silencing mechanism, slicer-dependent mRNA degradation or slicer-independent translation inhibition. Recent evidence indicates that P-bodies are essential for microRNA-mediated gene silencing and that RISC assembly and silencing occurs primarily within P-bodies. The P-body model outlines microRNA sorting and shuttling between specialized P-body compartments that house enzymes required for slicer –dependent and –independent silencing, addressing the reversibility of these silencing mechanisms. Detailed knowledge of the microRNA pathways is essential for understanding their physiological role and the implications associated with dysfunction and dysregulation.

## INTRODUCTION

MicroRNA (miRNA), originally discovered in *Caenorhabditis elegans,* is found in most eukaryotes, including humans [1-3]. It is predicted that miRNA account for 1-5% of the human genome and regulate at least 30% of protein-coding genes [4-8]. To date, 940 distinct miRNAs molecules have been identified within the human genome [9-12] (http://microrna.sanger.ac.uk accessed July 20, 2010). Although little is currently known about the specific targets and biological functions of miRNA molecules thus far, it is evident that miRNA plays a crucial role in the regulation of gene expression controlling diverse cellular and metabolic pathways [13-19].

MiRNA are small, evolutionary conserved, single-stranded, non-coding RNA molecules that bind target mRNA to prevent protein production by one of two distinct mechanisms. Mature miRNA is generated through two-step cleavage of primary miRNA (pri-miRNA), which incorporates into the effector complex RNA-induced silencing complex (RISC). The miRNA functions as a guide by base-pairing with target mRNA to negatively regulate its expression. The level of complementarity between the guide and mRNA target determines which silencing mechanism will be employed; cleavage of target messenger RNA (mRNA) with subsequent degradation or translation inhibition Fig. (**1**) [1, 20]. 

There is a general understanding of miRNA function but the mechanistic details of miRNA biogenesis and gene silencing are still unclear. This new and exciting field of molecular biology continues to advance, having profound implications in medicine. Although the biological function of identified miRNAs may be unknown, examination of the expression profiles of these molecules provides information on their regulation and function. Such observations have indicated that miRNA expression profiles are altered in specific tumors, implying that miRNA may be involved in development of cancer and other diseases [21-27]. Despite the limited knowledge of these molecules, basic expression profiling is proving to be clinically relevant to cancer diagnosis, progressions and outcome [24, 28-31].

This review will explore recent advances in human miRNA biogenesis and function to propose a working model of miRNA gene silencing. Secondly, it will examine miRNA involvement in cancer development and the medical implications. 

## MICRORNA – IN THE GENOME

There are many classes of small endogenous RNA molecules, such as small transfer RNA (tRNA), ribosomal RNA (rRNA), small nucleolar RNA (snoRNA), small interfering RNA (siRNA) and microRNA (miRNA). MiRNA and siRNA are biochemically and functionally indistinguishable. Both are 19-20 nucleotides (nt) in length with 5’-phosphate and 3’-hydroxyl ends, and assemble into RISC to silence specific gene expression [32-34]. Therefore, these molecules are distinguished based on their respective origins. MicroRNA is derived from the double-stranded region of a 60-70nt RNA hairpin precursor while siRNA is generated from long double-stranded RNA (dsRNA) [20, 32, 34]. Originally all small RNA that mediated post-transcriptional gene silencing *via *RISC was referred to as siRNA regardless of origin, however now it is common procedure to distinguish between miRNA and siRNA.

MiRNA precursors are commonly found in clusters through many different regions of the genome, most frequently within intergenic regions and introns of protein-coding genes. Historically these regions were referred to as “junk DNA” because their function was unknown. The discovery of miRNA genes in this vast component of our genome implies ‘junk DNA’ is not useless as originally thought. MiRNA precursors are less commonly found within exons of transcripts and in antisense transcripts [35, 36].

MiRNA transcriptional units and their regulation vary with gene loci. Intronic miRNAs located within a host gene, in the same orientation are transcribed along with primary transcript by the same promoter [36, 37]. In contrast, intergenic miRNA presumably rely on their own promoters [35, 36, 38]. The nature of intergenic miRNA transcriptional units is just beginning to unfold. Recent genomic analysis indicates that primary miRNA precursors are long polycistronic transcripts that are similar to mRNA in that they have distinct 5’ and 3’ boundaries, 7-methyl guanylate (m7G) caps and poly(A) tails [12, 38-41]. 

The discovery of intergenic miRNA and protein-coding intronic miRNA coupled with work of Lee *et al*. (2004) indicates that the majority of miRNA are transcribed by RNA polymerase II (pol II) [38, 42-44]. Pol II is the catalytic component of the complex of protein responsible for transcribing DNA into mRNA [45-47]. However, miRNA can also be transcribed by RNA polymerase III (pol III) which specifically synthesizes small non-protein coding RNAs that are linked to regulating cell cycle and growth [48-57]. Investigation of the miRNA cluster C19MC on the human chromosome 19 transcribed by pol III revealed that it was interspersed with Alu repeats, a characteristic of pol III transcription [57, 58]. Extrapolating this observation Borchert *et al*. suggest that pol III transcription of miRNA might be more prevalent than originally thought, noting that at least 50 additional human miRNA loci are among repetitive elements associated with pol III transcript [57]. A recent study by Gu *et al*. (2010) identified 68 new miRNAs that appear to be transcribed by an Alu-dependent pol III mechanism [59].

The synthesis of miRNA by pol II and pol III implies that miRNA is a fundamental regulatory element generated from diverse loci within the human genome, which are involved in controlling gene expression essential for normal cellular function.

Regulation of miRNA expression remains largely unknown. MiRNA may be expressed in a tissue- or developmental- specific manner [19, 20, 60-62]. Primary miRNA transcripts generated by pol II are presumably regulated similar to protein coding transcripts. MiRNA expression can be controlled by transcription factors and possibly other miRNA in response to a variety of endogenous and exogenous stimuli [63-69]. Regulation of the multiple processing steps in miRNA biogenesis can also influence expression. Proteins such as HnRNPA1, SMAD1 and SMAD5 have been shown to interact with miRNA precursors and regulate their subsequent processing to mature miRNA [70, 71]. Regulatory proteins can also bind mature miRNA to direct their degradation, preventing their expression. Lin28 is an example of such a regulatory protein, it binds let-7 miRNA and targets its degradation [72]. 

It is estimated that 10% of miRNA expression is controlled through DNA methylation [73]. Additional evidence supports miRNA regulation in response to hypoxia, hormonal and dietary changes [63-65]. MiRNA regulation becomes even more complicated with the discovery of intricate feedback loops, which will be discussed in detail later [67-69, 74]. 

## MICRORNA BIOGENESIS – NUCLEAR FIG. (2)

Human miRNA biogenesis is a two-step process, with both nuclear and subsequent cytoplasmic cleavage events performed by two ribonuclease III endonucleases, Drosha and Dicer [75-81]. The miRNA gene is transcribed to produce a primary miRNA (pri-miRNA) that is processed into a precursor miRNA (pre-miRNA) and subsequently miRNA duplex (miRNA:miRNA*, passenger strand designated with asterisk) which ultimately releases mature miRNA [44]. MiRNA origin and size appear to determine what nuclear pathway the miRNA will proceed through. 

The first determinant is the origin of the miRNA, being either intergenic or coding-intronic [36, 82].

### Intergenic microRNA

Intergenic miRNA are transcribed by pol II or pol III producing a pri-miRNA which is a large stem-loop structure with single-stranded RNA extensions at both ends [42, 57, 83]. Only those pri-miRNA with the appropriate stem length, a large flexible terminal loop (≥ 10bp) and the capability of producing 5’ and 3’ single-stranded RNA overhangs will be efficiently processed and mature into functional miRNA [80, 84-88]. The maturation process begins with the nuclear cleavage of pri-miRNA by a protein complex known as “the microprocessor”, which is comprised of the RNase III endonuclease Drosha and the double-stranded RNA-binding protein DiGeorge syndrome critical region gene 8 (DGCR8) [75, 77, 78, 80, 81]. Recent evidence indicates unique cross-regulation between Drosha and DGCR8 is important for control of miRNA biogenesis. In this cross-regulation DGCR8 stabilizes Drosha *via *protein-protein interaction and assists in controlling Drosha protein levels [89]. Conversely, Drosha in the context of the microprocessor negatively regulates DGCR8 mRNA post-transcriptionally by cleaving an 88 nt hairpin located in the 5’ untranslated region (UTR) which destabilizes the transcript [89, 90]. Additional microprocessor-associated proteins are required for processing of particular pre-miRNAs, such an example is miR-18a which requires the protein factor hnRNPA1 [71, 91]. The most current processing model suggest that DGCR8 recognizes the pri-miRNA at the ssRNA-dsRNA junction and directs Drosha to a specific cleavage site ~11 base pairs (bp) from the junction on the stem where Drosha cuts to liberate a ~ 60-70 bp miRNA hairpin precursor (pre-miRNA) [78, 80, 81, 83, 86, 92, 93]. The pre-miRNA has one end of the mature miRNA defined by the cut from Drosha and contains the mature miRNA in either the 5’ or 3’ arm [76]. 

### Coding-Intronic microRNA

In contrast, miRNA located within an intron of a protein coding gene is transcribed by pol II as part of the pre-mRNA [36]. Evidence supports two possible miRNA excision processes which may be occurring simultaneously or independently [94]. Initial investigation determined that introns are excised out of the pre-mRNA and debranched by spiceosomal components [95, 96]. It is unclear whether the splicing process releases pre-miRNA that proceeds to nuclear export for maturation within the cytoplasm or if a pri-miRNA is released acquiring a secondary stem-loop structure and proceeding like intergenic pri-miRNA to be processed by the microprocessor complex [95, 96]. An additional hypothesized nuclear pathway might be possible for those small (~50-200nt) excised debranched introns containing miRNA, which have the structure to support hairpin formation. Recently such introns, referred to as mirtrons, have been discovered in invertebrates and mammals [97-99]. In invertebrates mirtrons bypass microprocessor cleavage, entering the miRNA maturation pathway during nuclear export to proceed through cytosolic processing. Although mammalian mirtrons have been identified it is unclear whether they actually proceed *via *the same miRNA maturation/functional pathway suggested by the evidence from invertebrates. 

The alternate miRNA excision hypothesis is that pre-mRNA splicing is not a requirement for pri-miRNA processing. In this hypothesis miRNA are excised after the splicing commitment has been made but prior to intron excision. Splice sites in the pre-mRNA are assumably marked and tethered by a splicing commitment complex permitting the microprocessor to selectively excises and liberate pre-miRNA, while splicing continues as normal to produce mature mRNA. This proposes that the microprocessor has an alternate recognition method from that described with intergenic pre-miRNA processing which is yet to be investigated [94, 99, 100].

### MicroRNA Nuclear Export

Subsequent pre-miRNA (and possibly mirtron) processing occurs in the cytoplasm. Pre-miRNA assembles into a complex with the nucleocytoplasmic transporter factor Exportin-5 and RanGTP, which prevents nuclear degradation and facilitates translocation into the cytoplasm [101-105]. The Exportin-5/RanGTP nuclear export pathway can support mirtron transfer to the cytoplasm in some species including *Xenopus laevis* oocytes and *Drosophila* but it remains unclear whether this occurs in mammals [97, 102, 106]. It is possible that unidentified factors participate in mirtron nuclear export.

## MICRORNA BIOGENESIS – CYTOPLASMIC FIG. (3)

The cytosolic component of miRNA maturation revolves around the RNase III endonuclease Dicer, which has been found in all eukaryotes examined to date except budding yeast [107]. Humans have a single isoform of Dicer that functions in both the siRNA and miRNA pathways [108, 109]. In contrast, *Drosophila* have separate isoforms of Dicer for the siRNA pathway and miRNA pathways [110, 111]. It is unknown how human Dicer distinguishes between siRNA and miRNA pathways. Dicer is a multi-domain protein located within the cytoplasm and/or the rough endoplasmic reticulum which is comprised of a N-terminal ATPase/Helicase domain, DUF283 (domain of unknown function), PAZ (Piwi/Argonaute/Zwilli) domain, and two tandem RNaseIII nuclease domains (RNase IIIa and RNase IIIb) located at the C-terminal followed by a dsRNA-binding domain (dsRBD) [112]. The PAZ domain, RNase III nuclease domains and the dsRBD are involved in the binding and cleavage of dsRNA [107, 113-116]. However, the function of the ATPase/Helicase domain and the DUF283 domain are unknown. Dicer characteristically cleaves dsRNA in an ATP independent manner but it has been suggested that ATP might be required for enzyme turnover assisting in product release and/or protein conformational changes [107, 117]. 

### Single Cleavage Pre-miRNA Processing

In one proposed model pre-miRNA translocates to the cytoplasm and is incorporated into the performed pre-miRNA processing complex composed for Dicer, the human immunodeficiency virus transactivating response RNA-binding protein (TRBP) and Protein Kinase R-activating protein (PACT) [1, 109, 118, 119]. This complex is the core of the RISC-loading complex [120]. The PAZ domain of Dicer recognizes the 2 nt 3’-overhang of the pre-miRNA and initiates binding [107, 113, 114]. The distance between Dicer’s PAZ and RnaseIII domains is used as a ruler to determine the exact cleavage site on the pre-miRNA [1, 113]. Intermolecular dimerization of Dicer’s two RNaseIII domains forms a single processing center with two catalytic sites that cleaves the single strand opposite that previously cleaved by Drosha in the pre-miRNA. The resulting product is a double stranded miRNA duplex with 3’ overhanging ends [113]. 

A recent model proposed by Cifuentes *et al*. (2010) centers around the Argonaute 2 protein (Ago2), also known as eukaryotic translation initiation factor 2C2 (eIF2C2) [121]. Ago2 contains a Piwi domain and a PAZ domain that possesses endonuclease activity [122-124]. This novel step-wise model describes a pre-miRNA processing pathway that is Ago2-dependent and Dicer-independent. Upon Ago2 pre-miRNA binding the endonuclease cleaves the passenger strand of the precursor hairpin 10 nucleotides upstream of the guide strand 5’end which facilitates unwinding. Those remaining nucleotides around the cleavage site that are not protected by Ago2 binding subsequently undergo polyuridylation and nuclease-mediated trimming to generate mature miRNA.

### Double Cleavage Pre-miRNA Processing

An additional Ago2-dependent pre-miRNA processing model proposed by Diedrichs and Haber (2007) suggests Dicer, TRBP and Ago2 form a protein complex that recognizes and binds the pre-miRNA through the PAZ domain of Dicer and/or Ago2. Ago2 then cleaves a single-strand of the pre-miRNA 11-12 nucleotides from the end of the on the 3’arm to generate a nicked hairpin structure referred to as “Ago2-cleaved precursor miRNA” or “ac-pre-miRNA”. The ac-pre-miRNA is the Dicer substrate cleaved to generate the double stranded miRNA duplex. 

There is evidence that Dicer, TRBP, PACT and Ago2 can all interact but no group has yet to describe a processing complex composed of all proteins. It appears the key players have been identified but the exact protein interactions remain unclear. Throughout the stages of miRNA biogenesis, proteins dissociate and re-associate with the active complexes, making it difficult to determine where in the miRNA pathway they are active. There is a consensus within the literature that TRBP associates with Ago2 and Dicer to play an integral role in the miRNA pathway [109, 118, 125, 126]. However, the exact function remains debated. Two studies performed by Haase *et al*. 2005 and Chendrimada *et al*. 2005 are in conflict [127]. Hasse *et al. *proposes that TRBP is essential for Dicer cleavage of pre-miRNA and the subsequent transfer to Ago2 for assembly into RISC. In contrast, Chendrimada *et al*. believe TRBP is only crucial for miRNA assembly into RISC. Additionally, an independent study has shown that TRBP can regulate Dicer protein levels *via *direct protein-protein interaction [128]. Further investigation is required to clearly define TRBP’s function. 

Interestingly, TRBP inhibits the interferon-induced dsRNA-activated protein kinase R (PKR) pathway and its associated protein PACT, which activates the PKR pathway [1, 129-132]. Activation of the PKR pathway inhibits general protein synthesis throughout the cell and induces the production of interferons, which mediate anti-proliferative and pro-apoptotic activity [133-135]. It appears that TRBP and PACT function to prevent cytoplasmic pre-miRNA activation of the PKR pathway and/or regulate PKR phosphorylation which controls components of the miRNA functional pathway [1, 119, 127, 136].

## RISC ASSEMBLY

Double stranded microRNA is generally a transient imperfect duplex molecule consisting of a passenger strand and a mature microRNA strand (also referred to as guide strand) commonly denoted miRNA:miRNA*, where the passenger strand is designated miRNA* [20, 33, 137]. However, some miRNA duplexes are near-perfect (extensive base-pairing in RNA duplex) [138]. Ultimately, the RNA duplex is unwound and the single strand mature miRNA is incorporated into the protein complex RISC to function as a guide, directing the silencing of target mRNA [20]. RISC assembly and activation has mainly been studied in *Drosophila* with relation to near-perfect base pair matches between the guide and passenger strands of the miRNA duplex. The RISC assembly model from *Drosophila* proposed ATP-dependent unwinding of the duplexes which enables the mature single-stranded guide to load into Ago2 of the RISC complex resulting in its activation [139, 140]. 

### *Drosophila* RISC Loading

In the *Drosophila* model of RISC loading, the passenger strand of the miRNA:miRNA* duplex is selected based on the thermodynamic stability of the 5’end. The strand with the most unstable 5’end will become the miRNA guide [141, 142]. In rare instances mature miRNA can form from both strands of a miRNA duplex [126, 143-145]. The process of miRNA duplex unwinding and subsequent degradation is similar to siRNA, where Ago2 cleavage of the passenger strand releases single strand mature miRNA to function in RISC [19, 20, 33, 60-62, 76, 126, 139-142, 146, 147]. Whether passenger strand cleavage occurs prior to RISC loading is unclear. The model proposed by Matranga *et al*. (2005) suggests that RNA duplexes (siRNA and miRNA) are loaded into Ago2 of RISC, which then cleaves the passenger strand, leaving the guide strand bound to Ago2. Single strand mature miRNA bound to Ago2 facilitates the RISC activation. However, the typical mismatch pairs within a miRNA:miRNA* duplex are thought to prevent the Ago2 cleavage-assisted mechanism and ultimately propel a “bypass mechanism”. The bypass mechanism is a slower process that ultimately results in the passenger strand dissociating from the guide strand although the mechanism remains unclear. 

### Human RISC Loading

In humans there are eight classes of RISC complexes, which are based on protein composition centered around the four argonaute proteins, Ago 1-4 [148]. Genomic analysis indicates that argonaute proteins have evolved from translation initiation protein, hence the synonym eukaryotic translation initiation factor [149]. Ago2 is the only argonaute protein with “slicer” activity capable of catalyzing cleavage of mRNA. However, all four Ago proteins associate with miRNA and appear to function in gene silencing. It is unclear whether specific Ago proteins are associated with a particular silencing mechanism. The core components of the RISC loading complex are Dicer, Ago2, PACT and TRBP [119, 126, 148]. RISCs that load miRNA are designated microRNA containing ribonucleoprotein complex (miRISC or miRNP). 

The mechanism of human miRNA RISC assembly is unclear. Hypotheses are mainly based on the *Drosophila* model. The mechanism of duplex unwinding is unknown, however evidence indicates the process is ATP-independent in humans [125, 126]. There are many hypotheses of duplex unwinding: Dicer could cleave the passenger strand, initiating unwinding and releasing the mature single strand miRNA that could be captured by Ago2. Alternatively, Ago2 could cleave the passenger strand of a loaded duplex, keeping the miRNA guide. Duplex unwinding also could occur simultaneously with conformational changes in RISC during assembly or by an unidentified helicase [126]. A recent study suggests that RNA Helicase A (RHA), also referred to as DHX9 or NDHll, is responsible for RNA duplex unwinding associated with RISC activation in the human miRNA pathway [150]. RHA is a DEAH-box protein with helicase activity capable of binding to ssRNA, dsRNA and dsDNA [150-152]. RHA is capable of unwinding RNA:RNA duplexes with 3’overhangs but the efficiency of RHA is unclear and is believed to be dependent on RHA’s interactions with associated proteins [150, 152, 153]. RHA interacts with Dicer, Ago2 and TRBP in a spatial arrangement optimal for unwinding RNA:RNA duplexes [150]. Additionally, the putative helicase MOV10 (Moloney leukemia virus 10 homolog) has been found to associate with some Ago2 RISC complexes but it remains unclear whether MOV10 is capable of unwinding RNA duplex structures [150, 154]. Interestingly, a very recent study by Yoda *et al*. (2010) suggests that Dicer cleavage and RISC assembly is uncoupled and ATP-dependent, which is in contrast to earlier studies [155]. Future studies are needed to confirm this finding.

## TARGET RECOGNITION

Activated RISC binds target mRNA through Watson-Crick base-pairing between the guide strand and the 3’UTR of the target [2, 3]. Target recognition relies heavily on base-pairing between the seed (residues 2-8 at the 5’end) of the miRNA guide [156-159]. The degree and nature of complementary sites between the guide and target appear to determine the gene silencing mechanism [20, 138]. Ago2 endonuclease cleavage is generally favored by near-perfect (extensive) base-pairing whereas the more common translation inhibition seems to require multiple complementary sites with only moderate (limited) base-pairing in each site [20, 138]. In animals, miRNA are generally 100% complementary in the seed but not over the whole miRNA which results in imperfect RNA hybrids with characteristic bulges [20, 93, 160, 161]. Binding at the seed is important for the thermal stability of the interaction, which contributes to miRNA specificity and activity [157, 162]. The phenomenon of G:U wobble (mismatch) base pairing in the seed was initially thought to be detrimental to miRNA function. miRNA:mRNA interaction can still occur in the presence of a G:U wobble but it effects specificity and activity of miRNA [156, 157, 163]. Subsequent investigation revealed this is not the case for all miRNAs. Mammalian miR-196 perfectly base pairs with its target HOXB8, with the exception of a G:U wobble involving U5 within the seed region and still efficiently down-regulates HOXB8 through mRNA cleavage [138]. 

## SILENCING

It is widely accepted that miRNA bind to their target mRNA and negatively regulate its expression. A single miRNA guide can regulate several mRNA targets and conversely multiple miRNAs can cooperatively regulate a single mRNA target [20]. However, the means of silencing remains unclear. Evidence supports two distinct silencing mechanisms; mRNA cleavage and translation repression, which can be defined as slicer-dependent and slicer-independent silencing [164-171]. Slicer activity refers to the endonuclease cleavage of target mRNA by Ago2 which requires extensive base-pairing between miRNA and mRNA target [124, 172-174]. Slicer-dependent and slicer-independent silencing mechanisms have one of two downstream effects, mRNA degradation or translation inhibition, both ultimately leading to down-regulation of gene expression. One significant difference between the downstream effects is reversibility, mRNA decay is an irreversible process. In contrast, translation inhibition is reversible because stable mRNA can be translated following elimination of translation repression [165, 174-177]. The following sections discuss the slicer-dependent and -independent silencing mechanisms.

### Slicer-Dependent Silencing Fig. (4)

MicroRNA-directed mRNA cleavage is catalyzed by Ago2, when the target and miRNA are extensively base-paired over regions including the seed and bases 10-11 of the guide [124, 138, 172, 174, 178, 179]. A single extensive complementary region is usually sufficient for cleavage. However, exceptions have been noted which suggest unidentified additional requirements might be necessary for slicer-dependent mRNA cleavage [86, 93, 157, 160, 174, 180]. Cleavage products are degraded by one of two processes responsible for bulk cellular mRNA degradation, both beginning with the deadenylation of the mRNA to remove the poly (A) tail [174, 181]. Subsequent degradation can occur *via *the exosome, which is a multi-protein complex with 3’-to-5’ exonuclease activity. Alternatively, the mRNA can undergo decapping by the enzymes Dcp1 and Dcp2 which facilitates 5’-to-3’ degradation by the exoribonuclease Xrn1p [174, 182].

### Slicer-Independent Silencing Fig. (5)

Multiple complementary sites with imperfect base-pairing create bulges in the RNA duplex inhibit the slicer activity of Ago2 [2, 3, 20, 174, 183, 184]. This does not affect the argonaute proteins ability to repress translation of the target mRNA [124, 172, 173, 185]. There is no distinct model of how miRNAs inhibit translation but it is clear that this can occur in various ways. Translation occurs in three stages: initiation, elongation and termination, which require coordination of multiple protein factors [186]. Experiments performed with mammalian cells provided evidence supporting miRNA inhibition of translation at both the initiation and elongation stages [183, 187]. However the selection mechanism is unclear. Recent data suggests the promoter used to transcribe the target mRNA determines which translation repression mechanisms will be employed [188]. Translation can also be regulated indirectly by spatial separation of components, such that miRNA-targeted mRNA is sequestered away from translational machinery into cytoplasmic foci known as P-bodies (also known as processing bodies, GW bodies and Dcp bodies) [165, 189-194]. Additionally, miRNA can accelerate target mRNA deadenylation and decapping independently of slicer activity consequently effecting translation initiation efficiency and/or transcript stability [169, 195-197]. The latter effect will lead to target mRNA decay *via *the same exosome and Xrn1p enzymatic degradation as slicer-dependent silencing [169, 182]. Unresolved issues with slicer-independent mRNA degradation also suggest involvement of unknown alternative mRNA decay pathways. 

### P-Bodies

P-bodies are dynamic small cytoplasmic protein spheroid domains found in cells ranging from yeast to humans [164, 166]. A large number of studies investigating the formation and function of P-bodies have been conducted in yeast. Although these structures are similar in humans there are mammalian specific proteins that might relate to functional diversity. There are a number of similar cytoplasmic domains found in cells, including stress granules, exosomes and multivesicular bodies that contain some of the same protein components [198, 199]. However, there are a few protein markers that appear to be specific to P-bodies such as Dcp1, 4E-T, GE-1/hedls, p54/RCK and Xrn1 [199]. There is some controversy in the field with regards to the classification of these small cytoplasmic domains that relates to varying function, localization, size and contained proteins. This is partly due to the dynamic nature of these structures. P-bodies are affected by a variety of cellular factors including glucose level, osmotic pressure and cell proliferation [194]. Similarly, stress granules respond in number and size to environmental stresses such as temperature, infection, hypoxia and ultraviolet light [200]. 

Collective research indicates P-bodies are the functional site of reversible mRNA repression and mRNA decay which are mediated by a variety of mechanisms including miRNA-mediated gene silencing [189, 190, 201, 202]. P-bodies contain mRNA along with a variety of enzymes and factors required for processes such as mRNA decapping, deadenylation, RNA degradation and translational repression [164, 182]. Further investigation of the link between miRNA silencing and P-bodies revealed that the RISC effector proteins Ago 1-4 localize in P-bodies with passenger and guide strands miRNA duplexes [183, 184, 189, 190, 203-206]. This suggests that in addition to being the functional site for general cellular mRNA turnover, P-bodies are also the functional site of miRNA-mediated gene silencing. The role of P-bodies in miRNA silencing is actively being investigated. Recent studies have compiled data to develop a P-body model of miRNA-mediated gene silencing. 

### The P-Body Model Fig. (6)

P-bodies and miRNA function are crucial to each other. Evidence clearly indicates that P-bodies are essential for miRNA function as inhibition of P-body formation by depletion of GW182 (the major protein component of P-bodies) significantly impairs miRNA function [190, 203, 207]. Conversely, removal of the microprocessor complex inhibits P-bodies formation [164, 194, 205, 207, 208]. 

RISC assembly and activation occurs within the P-bodies, which is supported the co-localization of miRNA duplexes and Ago 1-4 proteins to GW182 [183, 189, 190, 203-206]. GW182 localization and protein associations suggest it is a major component of RISC, having functional involvement in slicer-dependent and slicer-independent silencing mechanisms [154, 189, 190, 203, 204, 209-212]. This model hypothesizes specialized compartments within P-bodies for RISC recruitment of silencing effector proteins, slicer-dependent silencing, slicer-independent silencing.

Slicer directed mRNA cleavage conceivably occurs in a specialized P-body compartment responsible for mRNA degradation [183, 184]. Enzymes involved in the mRNA degradation process such as deadenylation enzymes Ccr4, Not1, Pop2, decapping enzymes Dcp1, Dcp2 and nucleases Xrn1p and the exosome, reside within P-bodies [182, 191, 192, 202, 213]. Alternatively, slicer-independent mechanisms inhibit translation by various means. 

P-bodies house translational control enzymes and factors, notably p54, FMRP, Gemin5 and RAP55, required for miRNA mediated translational repression and/or directed mRNA storage [192, 204, 213-218]. Storage compartments must be isolated from degradation enzymes and have a mechanism to manage mRNA status, which would determine if transcripts get degraded or return to the cytoplasm for active translation [171, 219, 220]. On the other hand, mRNAs being actively translated by polyribosomes are targeted by miRISC in the cytoplasm to inhibit translation initiation and/or elongation [165, 175-177]. These mRNAs could then be directed to P-bodies for storage or degradation. MiRNA repression of actively translated mRNAs may explain why miRNAs and argonaute proteins are also found within the cytoplasm. 

### MicroRNA Silencing in the Nucleus?

The majority of data indicates that miRNA mediated post-transcriptional gene silencing occurs in the cytoplasm and P-bodies. However, the recently miRNA and argonaute proteins have been discovered in the nucleus of human cells and shown to repress gene expression of nuclear target RNA [221-225]. MiRISC could conceivably assemble and/or be imported into nuclease. A study in *Caenorhabditis elegans* identified an argonaute protein, NRDE-3 (nuclear RNAi defective-3), that imports siRNA to the nucleus [226, 227]. It is possible that humans have transporter proteins with similar function. 

The function of nuclear miRISC is unclear. However, the discovery designates nuclear non-coding RNA as a new class of miRNA targets. MiRNA may function in the nucleus to regulation gene transcription, prevent RNA export or affect target mRNA splicing. Perhaps nuclear miRNA, in conjunction with argonaute proteins, directs DNA methylation. Alternatively, miRNA could localize in the nucleus for modification. Nuclear A-to-I editing of miRNA by adenosine deaminase that act on RNA (ADAR) has been implicated in the regulation of miRNA processing [217, 228-231]. The scope of miRNA silencing remains unclear, as does a relationship between different silencing mechanisms.

## PROPOSED MICRORNA WORKING MODEL FIG. (7)

Upon stimulation miRNA is transcribed from the genome either as a component of pre-mRNA or a polycistronic primary miRNA transcript. Cleavage of the pre-mRNA by the spliceosome or microprocessor releases one of three possible miRNA precursor molecules, a mirtron, pri-miRNA or pre-miRNA. Pri-miRNA are processed into pre-miRNA by the microprocessor complex which cleaves one arm 11 bp from the ssRNA-dsRNA junction. Pre-miRNA (and possibly mirtrons) are exported to the cytoplasm *via *the Exportin-5/RanGTP pathway. In the cytoplasm pre-miRNA can mature into a miRNA duplex through two alternative pathways. The direct pathway is a single cleavage event performed by Dicer, which is associated with TRBP and PACT. In contrast, the indirect pathway is a two-step cleavage process carried out by a complex comprised of Dicer, Ago2 and TRBP. An initial Ago2 cleavage generates a nicked intermediated, ac-pre-miRNA, which acts as a substrate for Dicer. The miRNA duplex loads into Ago2 of the RISC loading complex that contains Dicer, TRBP, and PACT. If the miRNA duplex is extensively base-paired Ago2 will cleave the arm with the most stable 5’end, designated the passenger strand, triggering its degradation and leaving the guide strand bound to Ago2. However, in the instance that miRNA.

Active miRISC will direct mRNA awaiting translation or being translated to the recruitment compartment of a P-body, where the level of guide-target complementarity will regulate the recruitment of proteins required for slicer-dependent or slicer-independent silencing. Extensive guide-target base-pairing will relocate miRISC, with the bound mRNA target and recruited proteins, to the P-body compartment designated for slicer-dependent silencing where Ago2 will cleave the mRNA target. Subsequent mRNA decay will occur in the specialized mRNA degradation compartment at which time miRISC will return to the recruitment unit for ATP-dependent remodeling necessary for additional silencing. Alternatively, limited guide-target base-pairing will direct relocation to a compartment specific for slicer-independent silencing. In this unit mRNA will either associate with translation repression factors or be relocated for long-term mRNA storage or accelerated mRNA decay which releases miRISC for remodeling.

## MicroRNA IN CANCER

Cancer is a multistep process in which normal cells experience genetic changes that progress them through a series of pre-malignant states (initiation) into invasive cancer (progression) that can spread throughout the body (metastasis). The resulting transformed cellular phenotype has several distinct characteristics that enables cells to proliferate excessively in an autonomous manner; Cancer cells are able to proliferate independent of growth signals, unresponsive to inhibitory growth signals, evade programmed cell death (apoptosis) pathways, overcome intrinsic cell replication limits, induce and sustain angiogenesis, and form new colonies discontinuous with the primary tumor [232]. 

The dysregulation of genes involved in cell proliferation, differentiation and/or apoptosis is associated with cancer initiation and progression. Genes linked with cancer development are characterized as oncogenes and tumor suppressors. Oncogene products can be categorized into six groups based on their function; they can be transcription factors, growth factors, growth factor receptors, chromatin remodelers, apoptosis regulators or signal transducers [233]. Over expression of these gene products provide selective growth advantages that can drive tumor development. Oncogenes can be activated by genetic alterations that amplify the gene, alter promoters/enhancers to increase gene expression or alter protein structure to a permanent active state [233-235]. Conversely, tumor suppressor gene products have regulatory roles in biological processes. Loss or reduction of function of tumor suppressors results in dysregulation associated with cancer [236]. Recently, the definition of oncogenes and tumor suppressors has been expanded from the classical protein coding genes to include miRNA [24, 237]. MiRNAs play a vital role in regulating numerous metabolic and cellular pathways, notably those controlling cell proliferation, differentiation and survival [13-18, 238-240]. 

Dysregulation of miRNA expression profiles has been demonstrated in most tumors examined [24, 241]. However, the specific classification of miRNA as oncogenes or tumor suppressors can be difficult because of the intricate expression patterns of miRNAs. MiRNAs expression patterns differ for specific tissues and differentiation states, which poses two difficulties in classification [21, 22, 31, 242, 243]. It is not always clear if altered miRNA patterns are the direct cause of the cancer or rather an indirect effect of changes in cellular phenotype. Additionally, a single miRNA can regulate multiple targets [158]. This coupled with tissue specific expression could implicate a single miRNA as a tumor suppressor in one context and an oncogene in another. This is the topic of the next section.

## MicroRNAs AS TUMOR SUPPRESSORS & ONCOGENES

### Let-7

Let-7 miRNAs (let-7s) were one of the first miRNAs discovered in *Caenorhabditis elegans* [244]. Let-7s are highly conserved among invertebrates and vertebrates, including humans who have twelve let-7 genes encoding nine miRNAs [245]. Several let-7 genes have been mapped to regions within the human genome that are frequently altered or deleted in various cancers [245]. This discovery along with many other studies have implicated let-7 as a tumor suppressor. Two well-defined let-7 targets are the known oncogenes Ras and high mobility group AT-hook 2 (HMGA2).

Ras is a signal transducing GTPase that delivers signals from cell surface receptors to functional intracellular pathway effecting cell proliferation, growth, cytoskeleton organization, cell movements and survival [246-249]. Constitutively active Ras mutants (H-Ras, K-Ras and N-Ras) are found within a variety of human cancers including pancreas, colon, thyroid and lung [250]. Let-7s regulate the expression of Ras, K-Ras and N-Ras *via *3’UTR binding that inhibits translation [251]. Functioning as a tumor suppressor, let-7 mediates the suppression of Ras and the cellular processes it modulates [251-254]. 

HMG2A is non-histone architectural transcription factor that alters DNA conformation to direct transcriptional activation of a variety of genes that influence cell growth, differentiation, proliferation and survival [255]. HMG2A is undetectable in normal adult tissue but highly expressed in embryonic tissues, lung cancer and uterine leiomyomas [256-258]. Studies reveal that let-7s regulate HMG2A by destabilizing its mRNA through 3’UTR binding [21-23]. This regulation can explain the HMG2A expression pattern as let-7 exhibits a reciprocal expression pattern compared to HMG2A in embryonic and mature tissue [22]. Loss of let-7 HMG2A control promotes cell growth and proliferation [21, 22].

The let-7 family of miRNAs regulate numerous genes not all of which are as well defined as HMG2A and Ras. Recent data indicates that let-7s play a much larger role in controlling cell proliferation than initially thought as they have been shown to functionally inhibit numerous cell cycle regulators including c-myc, CDC25A, CDK6 and cyclin D2 [253, 259, 260].

### miR15 and miR16

The miR15/16 family of miRNAs has four members that are putative tumor suppressors. These members are organized in two distinct clusters, miR15a/miR16-1 located at 13q14.3 and miR15b/miR16-2 at 3q26 [144, 261, 262]. All four miRNAs share a 9-nucleotide seed region that targets the 3’UTR of the anti-apoptotic protein BCL2 for post-transcriptional repression [261, 263]. The oncogenic BCL2 protein, commonly over expressed in various tumors and hematopoetic malignancies, promotes cell survival by evading apoptosis [264-266].

The miR15a/16-1 miRNA cluster was found to be a frequent deletion site in B cell chronic lymphocytic leukemia (CLL) patients [262]. Evidence suggests that in this instance the absence of miR15a/16-1 is associated with a loss of regulation resulting in elevated BCL2 protein levels [261-263, 267]. BCL2 functions to block apoptosis by inhibiting mitochondrial cytochrome C release necessary to activate the caspase pathway of enzymes responsible for directing programmed cell death [268, 269]. Overall, the consensus is that normal miR15a/16-1 expression induces intrinsic apoptosis pathways by mediating BCL2 repression.

A recent study focusing on miR15b/miR16-2 expression found similar results as those observed with miR15a/16-1 [263]. Additionally, evidence was provided that suggested miR15b/miR16-2 plays a role in increasing chemosensitivity. It is hypothesized that BCL2 repression *via *miR15b/miR16-2 reduces a cell’s sensitivity to drug-induced apoptosis [263, 270-272]. 

The miR15/16 family of miRNAs has numerous predicted targets in addition to BCL2. Computer algorithms and proteomic analysis predict a variety of targets linked to cell growth, cell cycle and apoptosis [267, 273]. However, it is unclear whether these are direct or indirect miRNA targets.

### miR-21

MiR-21 is a relatively new member of the oncogenic miRNA group. Initially found to be over expressed in human glioblastoma tumors, miR-21 was described as an anti-apoptotic factor predicted to down regulate genes associated with advancing apoptosis [274]. Subsequently, miR-21 over-expression was observed in a variety of human cancer, including those derived from breast, colon, liver, brain, pancreas and prostate [241, 274-279].

Recent studies have revealed that miR-21 down-regulate four tumor suppressors genes: mapsin, programmed cell death 4 (PDCD4), tropomyosin1 (TPM1) and phosphatase and tensin homolog (PTEN). Evidence suggests that miR-21 directly binds to the 3’UTR of the gene transcripts preventing their translation. MiR-21 repression of these tumor suppressor genes promotes cell transformation, tumor growth, invasion, and metastasis [275, 277, 280-282]. 

PTEN is a phosphatidylinositol phosphate (PIP) phosphatase that functions in conjunction with phosphoinositide 3-kinase (PI3K) to balance PIP3 levels that regulate the Akt pathway [283]. PTEN dephosphorylates PIP3 and PI3K phosphorylates PIP2 to maintain the crucial balance of PIP3 levels [Li, 2007 #8933, 284]. Translational repression of PTEN by miR-21 leads to an accumulation of PIP3 that over stimulates the Akt pathway, activating multiple downstream pathways that stimulate cell growth and survival [275, 280, 285]. Additionally, loss of PTEN is associated with an increase in focal adhesion kinase (FAK) activity that promotes cell migration and metastasis by increasing the expression of several matrix metalloproteases [275, 280, 286].

TPM1 is an actin binding protein involved in the control of anchorage-independent growth and cellular microfilament organization [287, 288]. It is hypothesized that miR-21 repression of TMP1 leads to cytoskeleton changes that promote neoplastic transformation, cell invasion and metastasis [282]. Similarly, repression of PDCD4 and mapsin are thought to promote cancer progression by promoting cell invasion and metastasis [277, 280, 281, 289]. There are multiple proposed mechanisms of PDCD4 and mapsin action, all of which are still under investigation. Both molecules are associated with apoptosis and regulation of the urokinase receptor (uPAR), which is involved in degradation of the extracellular matrix [280, 290-292]. Thus implicating a role in tumor progression and metastasis. 

### miR-17-92

The miR-17-92 miRNA cluster is the most complex. The sequence and organization of the miR-17-92 cluster is highly conserved among all vertebrates examined [293]. In humans, the cluster produces six mature miRNAs (miR-17, miR-18a, miR-19a, miR-19b-1, miR-20a and miR-92-1) from a polycistronic transcript generated from the third exon of the open reading frame C13orf25 at loci 13q31.3 [293, 294]. MiR-17-92 can be directly regulated by c-myc and E2F transcription factors (E2Fs) 1-3 but, E2F3 is thought to be the predominant regulator [67-69, 295]. Evidence supports a dual role for miR-17-92 as both an oncogene and a tumor suppressor. However, the miR-17-92 functional network is extremely complex and remains under extensive investigation. 

MiR-17-92 is most commonly thought of as an oncogene [296]. The 13q31.3 gene locus is a frequent site for gene amplification, which explains highly elevated levels of miR-17-92 miRNAs observed within a variety of lymphomas, lung cancer, colon, pancreas and prostate cancers [241, 294, 297-299]. Additionally, over expression of c-myc is commonly observed in human cancers, which would result in an increase in miR-17-92 expression [300-303]. MiR-17-5p and miR-20a of the miR-17-92 cluster bind to target sequences in the 3’UTR of E2F transcription factors to inhibit their translation [67-69].

E2Fs are critical regulatory components for apoptosis and cell proliferation [304-312]. E2F 1-3 are associated with the activation of genes required for G1/S phase progression in the cell cycle [305, 306]. However, isoform specificity is apparent. E2F1 is primarily involved in promoting apoptosis and E2F3 signals more specifically for proliferation [68, 69, 313]. The crucial balance of E2F isoform expression modulates apoptosis and proliferation in normal cells. E2Fs stimulate the transcription of their own genes and of their regulators, c-myc and the miR-17-92 cluster. This creates an E2F positive auto-regulatory feedback loop which is controlled by miR-17-92 in a negative feedback loop [67-69, 314, 315]. Additionally, c-myc regulates E2Fs and miR-17-92, establishing a double feed-forward loop [68, 316]. The oncogenic activity of miR-17-92 is hypothesized to promote tumorigenesis by selectively repressing E2F1. Over expression of miR17-92 would decrease E2F1 levels needed to induce apoptosis. This does not trigger the negative feedback loop because E2F3 is the predominant miR-17-92 regulator instead there is an increase in E2F3 levels that will stimulate proliferation.

Although the majority of data supports miR-17-92 as an oncogene there is some evidence that it also acts as a tumor suppressor. Coincidently, loss of heterozygosity and deletions at 13q31.3 have been observed in various cancers, including breast cancer, hepatocellular carcinoma, retinoblastoma and nasopharyngeal carcinomas [317-320]. Recent data from breast cancer cell lines shows that miR-17-5p of the miR17-92 cluster down regulates the proto-oncogenic transcriptional activator AIB1 (amplified in breast cancer 1), also known as SRC-3, TRAM1, NCOA3 and RAC3 [321, 322]. AIB1 is a coactivator that increases the activity of transcription factors and various steroid receptors, which are involved in breast cell proliferation, growth and hormone signaling [323-329]. In this cellular context miR-17-5p is a tumor suppressor that regulates proliferation, growth, survival, differentiation and anchorage-independent growth by inhibiting AIB1 translation [321-324].

## CONCLUSION

Since their discovery, miRNAs have provided a new perspective on regulation of gene expression. Extensive research over the last decade revealed that miRNA are more prevalent that originally thought, with 940 miRNA identified to date in humans and over 1000 predicted [9-11, 330]. Although, much more investigation is required to determine all miRNA targets, silencing mechanisms and networks it is accepted that miRNAs play an integral role in regulating an array of fundamental biological processes. Evidence indicates that miRNA dysregulation is associated with disease, notably cancer as miRNA can function as oncogenes and tumor suppressors. MiRNAs are currently being exploited to advance cancer diagnosis, classification, prognosis and treatment [30].

MiRNA profiling was originally done with glass slide microarrays [331-334]. New advances have lead to the development of bead-based technology, specifically bead-based flow cytometric expression and bead-array profiling which is highly accurate, specific and feasible to implement within a clinical setting [31, 335]. This technology, and other techniques including quantitative reverse-transcriptase polymerase chain reaction (qRT-PCR), can identify distinct miRNA expression patterns that can characterize specific tissue and disease states, distinguishing between subtypes of normal and malignant tissues [278, 336, 337]. MiRNA signature profiles can be used to determine prognosis [338-340], response to drug therapy [341], predict treatment efficiency [342, 343], race susceptibility [23] and patient susceptibility to cancer and metastasis [344-346]. This has been demonstrated with lung cancer where unique miRNA profiles can distinguish between normal lung and tumor tissue and correlate to patient prognosis [340]. Additionally, miRNA profiles can discriminate between normal and malignant B-cells in chronic lymphatic leukemia and could hypothetically be utilized to classify undifferentiated tumors to their organ of origin [344]. 

Initially miRNA profiling was conducted on samples extracted from tissues. However recent studies have found stable miRNAs in readily available body fluids including, serum [347, 348], plasma [349-351], urine [352] and saliva [353]. The source of the endogenous circulating miRNAs is unclear. The predominant hypothesis suggests that miRNAs (more likely pre-miRNAs) are circulating in exosomes that are shed from normal or tumor-derived cells [354]. Containment of miRNAs (pre-miRNA) within exosomes would protect the molecules from degradation and explain the molecules stability, even in harsh experimental conditions [355, 356]. However, a combination of other theories collectively suggest it is possible that the circulating miRNAs are from lysed cells and their stability is due to binding of DNA, proteins and or lipids that protect them from degradation [355, 357, 358]. 

Although the use of miRNAs as biomarkers in body fluids is exciting there are limitations that need to be overcome before there is wide-spread clinical application. More studies need to examine the ‘normal’ levels and diversity of miRNAs present with particular body fluids. It would appear classification of miRNAs would be necessary to account for different miRNA profiles that may arise depending on race, gender and age. The effects of cancer treatment, whether it be chemotherapy, surgery, radiation or a combination of these methods, should be investigated as it will presumably change miRNA profiles. One particular study showed specific reduction of miR-184 plasma levels following surgical resection of squamous cell carcinoma of the tongue [359]. Additionally, the mechanism of miRNA released into these body fluids needs to be characterized. Within the clinical setting the technological approach needs be standardized as a variety of handling and processing factors can results in dramatics changes in miRNA profiles [360]. Establishment of universal profiling approaches for specific samples (i.e. tissue, serum, plasma, urine, saliva) that addresses collection, extraction (sample purity), storage (frozen vs. fresh), profiling technique (noting RNA amount), normalization/standardization and statistical comparison.

Further research exploring the outcome of therapeutic treatment associated with different miRNA profiles could provide valuable information to advance drug regime selection. A recent study of patients with colon adenocarcinoma receiving fluorouracil-based chemotherapy revealed that high miR-21 expression is associated with a poor therapeutic outcome [361]. It is possible other chemotherapy drugs might have a better outcome in comparison to fluorouracil. As we gain understanding of specific miRNA mechanisms and function it could become evident that certain chemotherapeutic drugs might be favorable based on their mode of action in relation to a particular miRNA profile.

MiRNA modulation is emerging as a novel therapeutic target with the use of antagomirs. Antagomirs are chemically engineered oligonucleotides that inhibit miRNA target binding through competitive miRNA binding [14]. A variety of chemical modifications can be made to increase the stability and potency of antagomirs, including 2’-O-methyl modified ribose sugars [14], 2’-O-methoxyethyl-modified ribose sugars [13] and addition of an extra 2’-O, 4’-C methylene bridge in the ribose sugar [362]. A study conducted *in vivo* with mice utilized this novel therapeutic approach to inhibit miR-122 expression. Intraperitoneal injections administered the antagomirs to the liver successfully inhibited miR-122 liver expression, which decreased plasma cholesterol levels in normal and obese mice [13]. Thus miR-122 may be a good therapeutic target for hepatic metabolic diseases. This study suggests that antagomirs could be employed as a new treatment because they are long lasting, specific and have no significant associated toxicity. 

However, there are still many limitations associated with antagomirs as a therapeutic treatment for humans. Firstly, miRNA sequences and targets need to be further characterized and cataloged. MiRNAs can have multiple targets and a full understanding of their functional mechanisms would be required to design appropriate antagomirs. Although computational bioinformatics programs have made great improvements they still do not have a high enough accuracy to predict miRNA targets [363, 364]. Secondly, an effective method of delivery has yet to be developed for humans. High-pressure injection and electroporation can effectively delivery small RNAs, but they cause unwanted tissue damage [365-367]. A variety of viral vectors can efficiently introduce small RNA into cells [367-371]. While some vectors have been useful in animal studies they are not ideal for human because they can elicit an immune response that deflates their effectiveness [371-373]. Vectors that integrate into the genome can cause further problems with mutations associated with genome insertion [374, 375]. Strategies exploring liposome/cationic encapsulation delivery methods are advancing efficiency however, they may also elicit an immune response [365, 376]. Progress is also being made in the expanding field of nanobiotechnology to develop a means to package and deliver RNA to specific sites [377-380]. Viral vectors are being modified to improve expression specificity and make them safer for human trials [374, 381]. 

The young field of miRNA research is continually expanding. Current and future investigation will hopefully provide the much need information to sufficiently characterize the targets and functional mechanisms of miRNAs. A deeper understanding of miRNA molecular pathways would provide great insight in the initiation and progression of cancer, among other diseases and is essential to advance therapeutic treatments.

## Figures and Tables

**Fig. (1). MicroRNA maturation and function.**
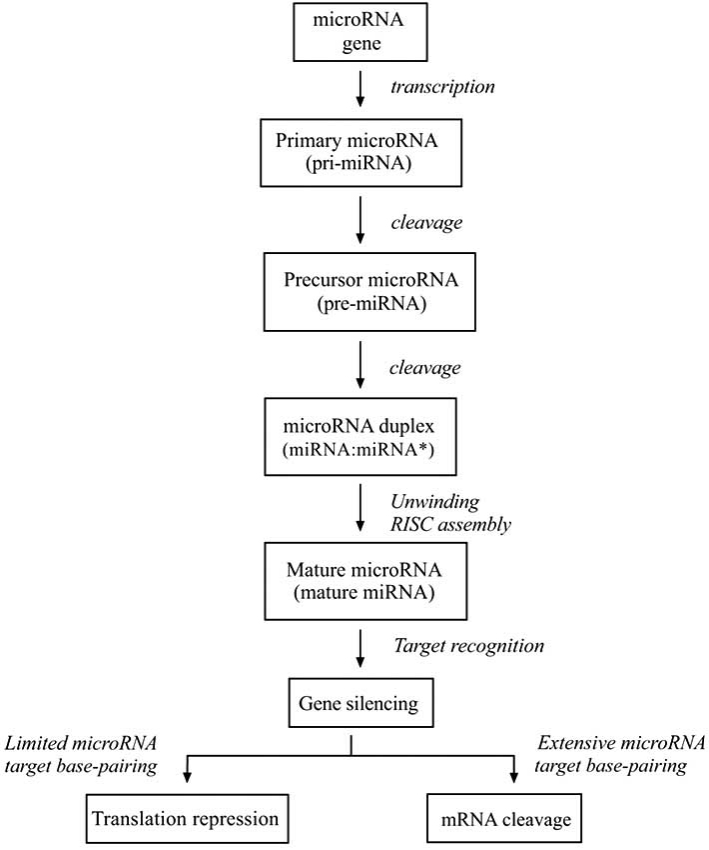
The miRNA gene is transcribed to generate a primary microRNA (pri-miRNA) precursor molecule that undergoes nuclear cleavage to form a precursor microRNA (pre-miRNA). The pre-miRNA is cleaved in the cytoplasm to create a microRNA duplex (miRNA:miRNA*, passenger strand designated with asterisk) containing the mature miRNA. The duplex unwinds and the mature miRNA assembles into RISC. The miRNA base-pairs with target mRNA to direct gene silencing *via* mRNA cleavage or translation repression based on the level of complementarity between the miRNA and the mRNA target.

**Fig. (2). Nuclear component of microRNA biogenesis.**
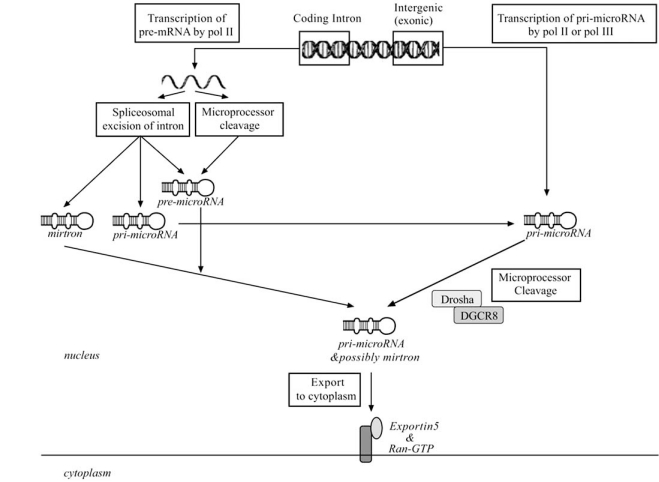
Intergenic miRNAs are transcribed by RNA polymerase II or III generating a primary miRNA (pri-miRNA) molecule, which is processed into a precursor miRNA (pre-miRNA) by the microprocessor complex comprised of DGCR8 and Drosha. Pre-miRNAs are exported to the cytoplasm in a nucleocytoplasmic transporter containing Exportin 5 and Ran-GTP. MiRNA within introns are transcribed as part of precursor mRNA (pre-mRNA) by RNA polymerase II. The miRNA sequence is excised from the pre-mRNA by spliceosomal components or the microprocessor to liberate a mirtron or a pre-miRNA that is exported. Alternatively, a primary miRNA (pri-miRNA) is released which undergoes microprocessor cleavage to generate pre-miRNA.

**Fig. (3). Cytoplasmic component of microRNA biogenesis.**
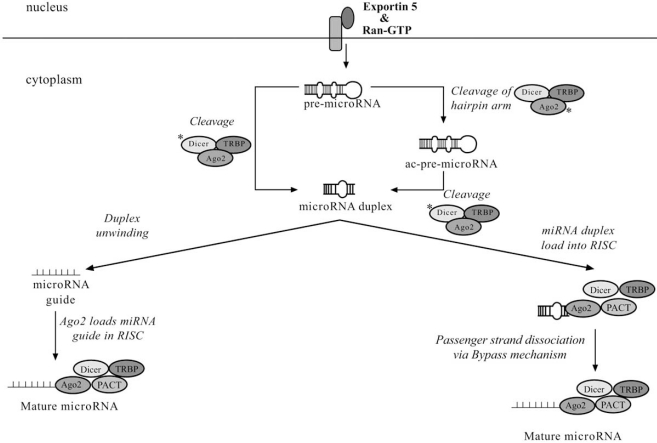
Pre-miRNA is cleaved by Dicer to generate miRNA duplex or by Ago2 to generate an Ago2-cleaved precursor miRNA” (ac-pre-miRNA) that subsequently acts as a substrate for Dicer. The asterisk (*) near a protein symbolizes it is responsible for the cleavage event. The miRNA duplex liberates the mature miRNA to assemble into RISC loading complex comprised of Ago2, TRBP, PACT and Dicer. The mechanism of mature miRNA release is unclear. Possible mechanisms involving RNA Helicase A (RHA), Dicer cleavage, Ago2 cleavage and uncharacterized proteins have been illustrated.

**Fig. (4). Slicer-dependent microRNA mediated gene silencing.**
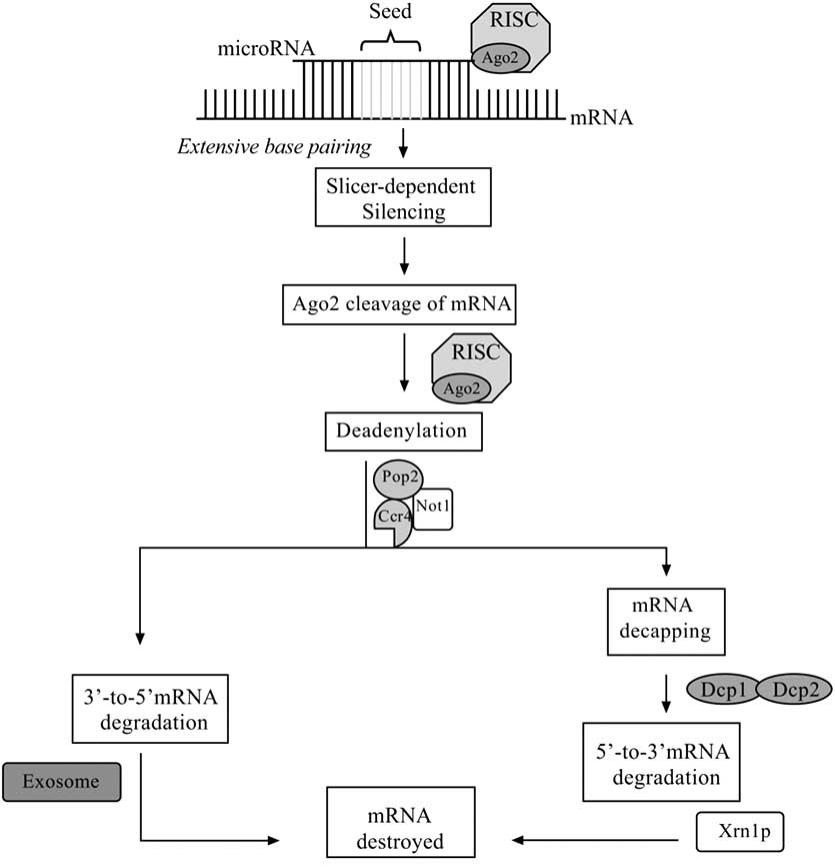
Extensive base-pairing between the miRNA guide and mRNA target permit Ago2 mRNA cleavage. Subsequently, mRNA is deadenylated by protein complexes comprised of Pop2, Ccr4 and Not1. mRNA is then degraded from 3’ to 5’ by the exosome complex or from 5’ to 3’ by the Xrn1p exonuclease following decapping by Dcp enzymes.

**Fig. (5). Slicer-independent microRNA mediated gene silencing.**
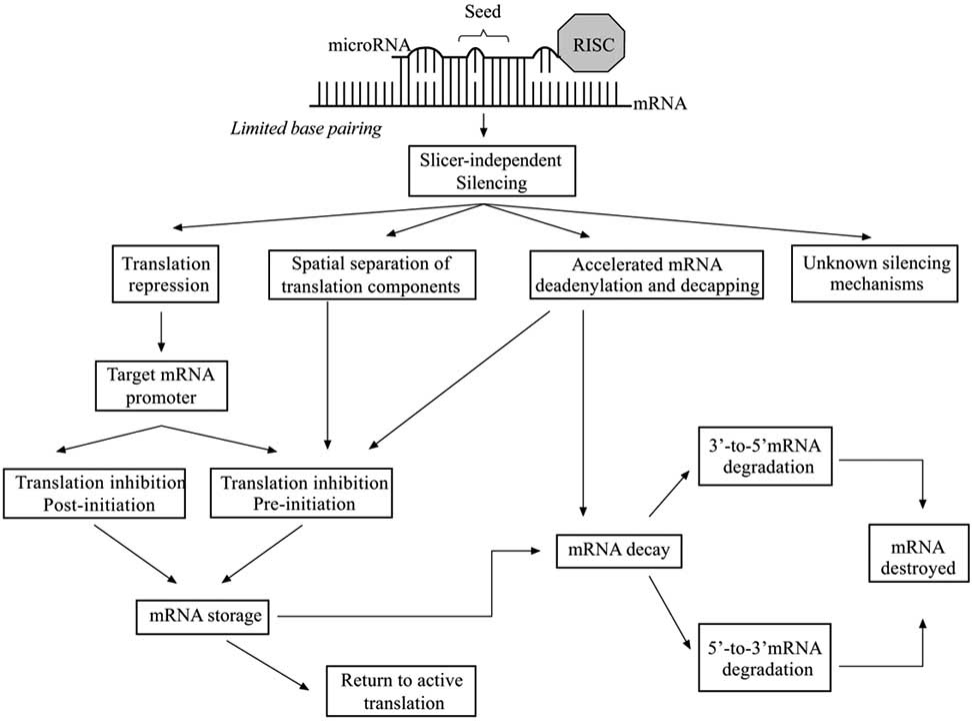
Limited base-pairing between the miRNA guide and mRNA target create bulges that inhibit Ago2 mRNA cleavage and propels slicer-independent gene silencing mechanisms. MiRNA can repress translation directly pre- or post- translation initiation which is determined by the target mRNA promoter. Alternatively, miRNA can repress translation indirectly by segregating mRNA away from ribosomes to cytoplasmic foci and accelerating deadenylation and decapping processes. Ultimately, mRNA can be isolated for storage or be targeted for decay *via* Xrn1p or exosome degradation. Stored mRNA can return to active translation or be targeted for degradation.

**Fig. (6). The P-body model.**
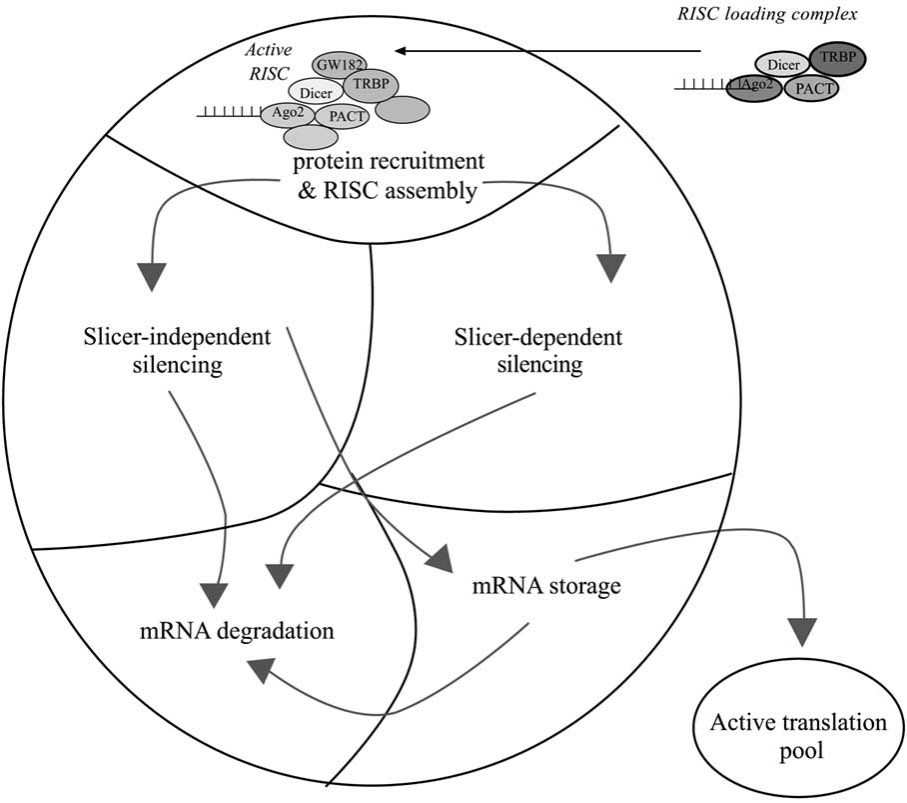
The RISC loading complex containing mature miRNA translocates to a P-body compartment specific for RISC assembly and activation. Upon target recognition the silencing mechanism is determine as slicer -depepndent or –independent, both having distinct functional compartments. Target mRNA will then be redirected for degradation or mRNA storage, which can be later returned to active translation or degraded.

**Fig. (7). Proposed microRNA working model.**
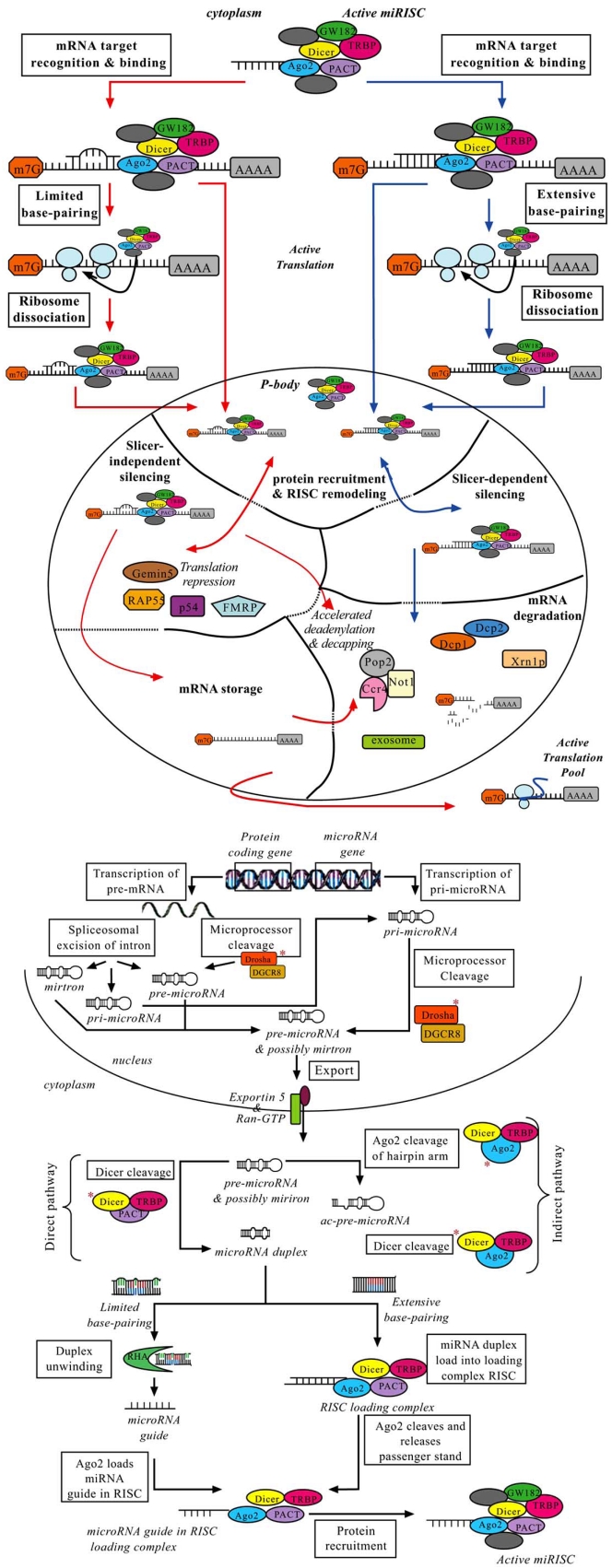
duplexes have mismatched pairs an Ago2 independent bypass mechanism will be employed. I hypothesize that this mechanism uses RNA helicase A to unwind the miRNA duplex which permits Ago2 of the RISC loading complex to load the arm with the least stable 5’end, the miRNA guide. Once the mature miRNA guide is bound to Ago2 additional RISC associated proteins, such as GW182, assemble to form the active miRISC which will seek out and bind its target mRNA.
